# Strong association of glaucoma with atherosclerosis

**DOI:** 10.1038/s41598-021-88322-4

**Published:** 2021-04-22

**Authors:** Xianqin Song, Peng Li, Yunfeng Li, Xinfeng Yan, Lin Yuan, Cong Zhao, Yi An, Xiaotian Chang

**Affiliations:** 1 Medical Research Center, The Affiliated Hospital of Qingdao University, Wutaishan Road 1677, Qingdao, 266000 Shandong People’s Republic of China; 2 Qingdao Engineering Technology Center for Major Disease Marker, Wutaishan Road 1677, Qingdao, 266000 Shandong People’s Republic of China; 3 Department of IT Management, The Affiliated Hospital of Qingdao University, Wutaishan Road 1677, Qingdao, 266000 Shandong People’s Republic of China; 4 Shandong Provincial Qianfoshan Hospital, Jingshi Road 16766, Jinan, 250014 Shandong China; 5 Department of Cardiology, The Affiliated Hospital of Qingdao University, Wutaishan Road 1677, Qingdao, 266000 Shandong People’s Republic of China

**Keywords:** Atherosclerosis, Glaucoma

## Abstract

Carbonic anhydrases (CAs) catalyze the synthesis of HCO_3_^-^ from H_2_O and CO_2._ The dysfunction of CAs leads to aqueous humor secretion and high intraocular pressure to cause glaucoma pathogenesis. Methazolamide (MTZ), a CA inhibitor, can effectively treat glaucoma by reducing aqueous humor secretion. We previously reported that carbonic anhydrase I (CA1), a CA family member, was highly expressed in atherosclerotic tissues of the aorta and stimulated atherosclerosis (AS) by promoting calcification. MTZ showed therapeutic and preventive effects on AS in a mouse model. The above findings suggest a relationship between AS and glaucoma. This study explored the possible association between AS prevalence and glaucoma prevalence and the therapeutic effect of MTZ on AS by analyzing medical records. Among 10,751 patients with a primary diagnosis of glaucoma, 699 (6.5%) were also diagnosed with AS. However, the incidences of AS in patients with keratitis and scleritis, which are also ophthalmic diseases, were 2.5% (206/8383 patients) and 3.5% (46/1308 patients), respectively. In the population without ophthalmic records, the AS prevalence was only 1.9% (99,416/5,168,481 patients) (all *p* values between each group were below 0.001). Among 152,425 patients with a primary diagnosis of AS, 1245 (0.82%) were also diagnosed with glaucoma. Among 199,782 patients with a primary diagnosis of hypertension (excluding AS), 1149 (0.57%) were diagnosed with glaucoma, and among 5,313,433 patients without AS or hypertension, 9513 (0.18%) were diagnosed with glaucoma (all *p* values between each group were below 0.001). Additionally, among 14 patients who suffered from both AS and glaucoma and were treated with MTZ to cure their glaucoma, 9 of them showed reduced low-density lipoprotein (LDL) levels, the main index of AS, within 3 months after medication use (2.81 ± 0.61 mmol/L vs. 2.38 ± 0.58 mmol/L, *p* = 0.039). The above findings demonstrated a strong relation between AS and glaucoma and suggested that AS patients with glaucoma were more likely to suffer from angle-closure glaucoma.

## Introduction

Glaucoma is the leading cause of irreversible vision loss worldwide and can be classified into 2 broad categories: open-angle glaucoma and angle-closure glaucoma, which can be classified as primary or secondary. Increasing intraocular pressure (IOP) is a primary cause of the development and progression of glaucoma^1,2^. Studies on the chemistry and dynamics of aqueous humor have identified sodium bicarbonate as the main constituent of this secretion. Carbonic anhydrases (CAs) catalyze the synthesis of HCO_3_^−^ from H_2_O and CO_2_, and the dysfunction of CAs leads to aqueous humor secretion and high IOP in glaucoma patients^3–5^. Methazolamide (MTZ), a CA inhibitor, is very effective in lowering IOP by reducing the rate of aqueous humor secretion^6–8^.

Atherosclerosis (AS) underlies most cardiovascular diseases (CVDs) and is accepted as a primary cause of mortality worldwide^9,10^. Recently, we found that carbonic anhydrase 1 (CA1), a CA family member, was overexpressed in aortic AS tissues. Our study also found that MTZ, an inhibitor of CA and an important drug in the treatment of glaucoma, significantly alleviated AS by inhibiting calcification in an AS mouse model^11^. Thus, it is highly possible that there is a strong association between glaucoma and AS because abnormal CA expression is involved in both glaucoma pathogenesis and AS calcification. It is also possible that MTZ may clinically alleviate AS in patients with both AS and glaucoma. Several studies have considered an intrinsic link between AS and glaucoma. A previous study evaluated the relationship between IOP and the risk of coronary artery calcification. The study found that increasing IOP was significantly associated with an increased prevalence of coronary artery calcium (CAC) and suggested that IOP was a predictive marker of CVD^12^. Another study found that serum lipid values, important markers of AS, could be predictive factors in primary open-angle glaucoma diagnosis^13^. These studies supported our hypothesis about the strong relation between AS and glaucoma. However, there are no clinical data to demonstrate a strong correlation between AS and glaucoma or the clinical characteristics of complications of the two diseases. There is no clinical evidence to support the therapeutic effect of MTZ on AS.

To verify the above hypothesis, we investigated the prevalence of AS in glaucoma patients and the incidence of glaucoma in patients with AS by analyzing medical records. To determine the potential therapeutic effects of MTZ on AS, this study also investigated the effects of MTZ on low-density lipoprotein (LDL) and high-density lipoprotein (HDL) levels, two biochemical indices of AS, in patients with both AS and glaucoma who received MTZ to treat glaucoma.

## Methods

This study was approved by the Ethics Committee of the Affiliated Hospital of Qingdao University. The study was performed in accordance with the ethical standards established in the 1964 Declaration of Helsinki and its later amendments. The Ethics Committee of the Affiliated Hospital of Qingdao University waived informed consent, as the current study only used retrospective clinical data from the Affiliated Hospital of Qingdao University.

**Figure 1:**
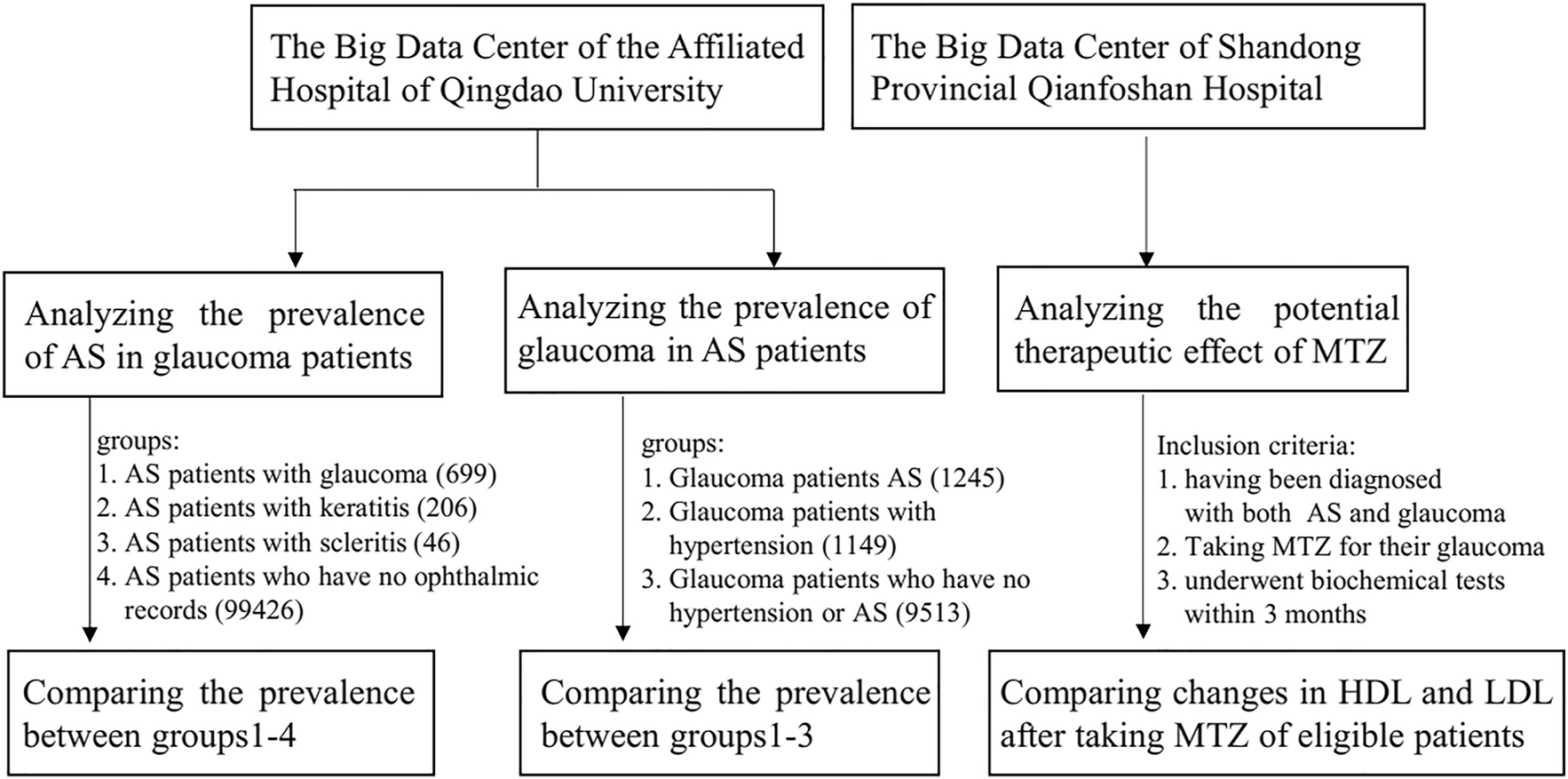
Flow chart for study design.

Atherosclerosis is a lipoprotein-driven inflammatory disorder leading to plaque formation at specific sites of the arterial tree, computed tomographic coronary angiography is a noninvasive imaging alternative that enables accurate evaluation of the luminal and outer vessel wall dimensions, high-risk plaque burden and morphology, and remodeling pattern^14^. The CT criteria for diagnosis of AS is that there are plaques in the vessels with lumen narrowing. To investigate the internal relationship between AS and glaucoma, a total of 6,114,142 cases were collected from the medical record database of the Affiliated Hospital of Qingdao University The clinical data of patients with cardiovascular diseases, including AS (152,425 cases), hypertension (199,782 cases) and ophthalmic diseases, including glaucoma (10,751 cases), keratitis (8383 cases) and scleritis (1308 cases), who were admitted to the hospital from June 2012 to April 2020 were collected and processed by Yiduyun, the Big Data Center of the Affiliated Hospital of Qingdao University. Each patient's sex, age, diagnosis, especially computed tomography (CT) angiography (including coronary artery, carotid, thoracic aorta and ventral aorta) findings and other information were obtained by consulting their medical records. The study design was as follows (Fig. 1).

To analyze the prevalence of AS in glaucoma patients, patients with a principal diagnosis of glaucoma were classified as the experimental group. Patients with a principal diagnosis of keratitis or scleritis as well as patients without ophthalmic records were classified as the control groups. Patients without ophthalmic records were referred to patients who had no record of ophthalmic consultation in the hospital. The data of patients who also suffered from AS in both groups were obtained by filtering their diagnoses using AS as the query term. Morbidity due to AS in both groups was determined and analyzed. Patients with a principal diagnosis of AS were classified as the experimental group. Patients with a principal diagnosis of hypertension and patients without hypertension or AS were classified as the control groups. Patients without hypertension and AS were referred to patients who had no hypertension or AS at the time of diagnosis. The prevalence rates of glaucoma in both groups were calculated and analyzed by filtering diagnoses using glaucoma as the query term.

To determine whether MTZ had a therapeutic effect on AS, we analyzed the data of patients who had been diagnosed with both AS and glaucoma and received MTZ for their glaucoma. The patients took 25 mg MTZ twice a day for two weeks and underwent a biochemical test within 3 months. LDL and HDL levels were measured in the patients during the test. Finally, 14 patients were eligible for the analysis comparing changes in HDL and LDL after taking MTZ; these eligible patients were identified by searching the Yiduyun database of Shandong Provincial Qianfoshan Hospital. The MTZ in this study was produced by Hangzhou Aoyibaoling Pharmaceutical (China). These patients did not take lipid-lowering drugs, such as statins, during the study period. The clinical characteristics of these 14 patients are shown in Table 1.

**Table 1:** The clinical records for the 14 eligible patients.

No	Patients number	Age	Gender	Time (period) of taking medicine	Test date before taking MTZ	Test date after taking MTZ
1	94	62	Female	20150609-20150612	2015-06-09	2015-08-11
				20150810-20150820		
2	375	29	Female	20180104-20180113	2017-12-21	2018-02-10
3	565	57	Male	2019-05-17	2019-05-13	2019-05-20
4	605	73	Female	2017-11-06	2017-11-03	2017-12-04
5	639	59	Male	2016-12-21	2016-12-20	2017-02-07
				2016-12-29		
6	685	66	Female	20180815-20180816	2018-08-14	2018-08-21
7	861	68	Male	2017-11-06	2017-11-03	2017-12-05
8	948	70	Male	20170207-20170216	2017-02-05	2017-04-09
9	297	80	Female	2017-03-15	2017-03-14	2017-05-31
				20190411-20190418		
10	385	54	Female	20180824-20180830	2018-08-23	2018-08-30
11	455	67	Male	20180324-20180403	2018-02-26	2018-04-01
12	657	82	Male	20180926-20181001	2018-09-26	2018-10-02
13	933	65	Female	2017-09-12	2017-09-11	2017-10-11
14	719	49	Female	2015-10-12	2015-10-11	2015-12-26
				2015-12-20		

### Statistical analysis

In this study, data entry was performed in EpiData 3.0 software (EpiData Association, Odense, Denmark). Double-entry and consistency tests were performed. SPSS 19.0 (SPSS Inc., Chicago, USA) software was employed for the statistical analyses. Means ± SDs were calculated for measurement data, and case numbers (constituent ratios) were calculated for enumerated data. Chi-square tests were performed to compare the incidence rates in the AS combined with glaucoma or glaucoma with AS groups with those in their respective control groups. Paired-samples T tests were performed to compare changes in LDL/HDL values before and after MTZ use. We also analyzed age compositions to compare morbidities among patients of different ages. *p* < 0.05 was considered statistically significant.

## Results

### Analyzing the prevalence of AS in glaucoma patients

**Figure 2:**
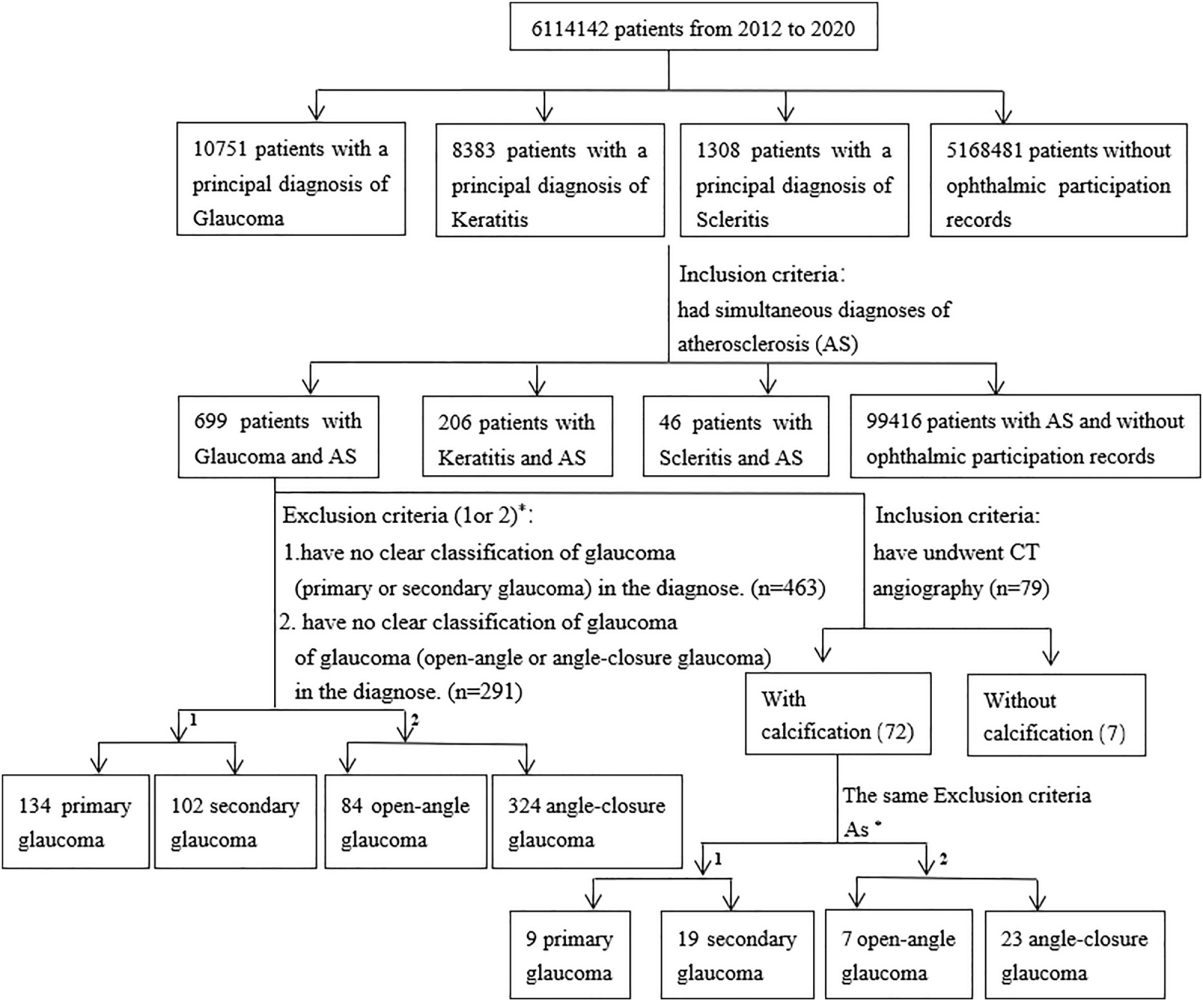
The analysis process of the morbidity of AS in patients with glaucoma.

In the present study, the data of patients with a principal diagnosis of glaucoma, keratitis, or scleritis and patients without ophthalmic records were analyzed, and the number of patients who suffered from AS was obtained by filtering diagnoses using AS as a query term. Then, the CT examination findings and diagnostic information of patients with both glaucoma and AS were derived, and the incidence rates of the different types of glaucoma were analyzed. The analysis process is shown in Fig. 2.

Among 10,751 glaucoma patients, 699 patients had simultaneous diagnoses of AS (6.5%). The incidence of AS in keratitis (excluding glaucoma) patients was 2.46% (206/8383 patients), and the incidence of AS in scleritis patients was 3.52% (46/1308 patients). The incidence of AS in the population without ophthalmic records was 1.92% (99,416/5,168,481 patients). The incidences of AS in the glaucoma groups were much higher than those in the other groups (all the *p* values between the groups were below 0.001).

Among the aforementioned 699 patients with both AS and glaucoma, 236 patients had diagnoses of primary glaucoma or secondary glaucoma. Among them, 134 (57%) were diagnosed with primary glaucoma, and 102 (43%) were diagnosed with secondary glaucoma. The remaining 463 patients were diagnosed with only glaucoma in the medical report. Six (4.48%) of the 134 patients with primary glaucoma were diagnosed with primary open-angle glaucoma, while the remaining 128 patients (95.5%) suffered from primary angle-closure glaucoma. The 102 patients with secondary glaucoma had no definitive description of open-angle or angle-closure glaucoma. Among the 699 patients, 408 patients were diagnosed with open-angle or angle-closure glaucoma. The remaining 291 patients were diagnosed with only glaucoma in the medical records. Among these 408 patients, 84 (20.6%) had open-angle glaucoma, and 324 (79.4%) had angle-closure glaucoma. This observation indicated that glaucoma patients with AS were more likely to suffer from angle-closure glaucoma.

Seventy-nine of the 699 patients with concurrent diseases underwent arterial CT angiography, including 36 cases of coronary artery angiography, 32 cases of head and neck angiography, and 11 cases of aorta angiography. Angiography examination showed that 72 patients (91.1%) had varying degrees of calcification. Among these 72 patients, 28 were diagnosed with primary glaucoma (9 patients or 32%) or secondary glaucoma (19 patients or 68%), and 30 patients were diagnosed with open-angle (7 patients or 23%) or angle-closure glaucoma (23 patients or 77%). This observation indicates that AS patients with arterial calcification may be prone to secondary glaucoma and angle-closure glaucoma.

The age composition of each group was also analyzed. A total of 426 patients (60.9%) among the 699 patients with both glaucoma and AS (group 1) were more than 65 years old. In contrast, 57.8% (119/206 cases) of the patients diagnosed with both keratitis (excluding glaucoma) and AS (group 2), 56.5% (26/46 patients) of the patients diagnosed with both scleritis and AS (group 3), and 56.5% (56,189/99,416 patients) of the patients with no ophthalmic participation records (group 4) were more than 65 years old. The differences in the age compositions between group 1 and groups 2 and 3 were not significant (*p* = 0.431/0.661). The *p* value of the difference between groups 1 and 4 was 0.019. The above observations suggest that glaucoma complicated with AS was more likely to occur in people older than 65 years.

### Analyzing the prevalence of glaucoma in AS patients

**Figure 3:**
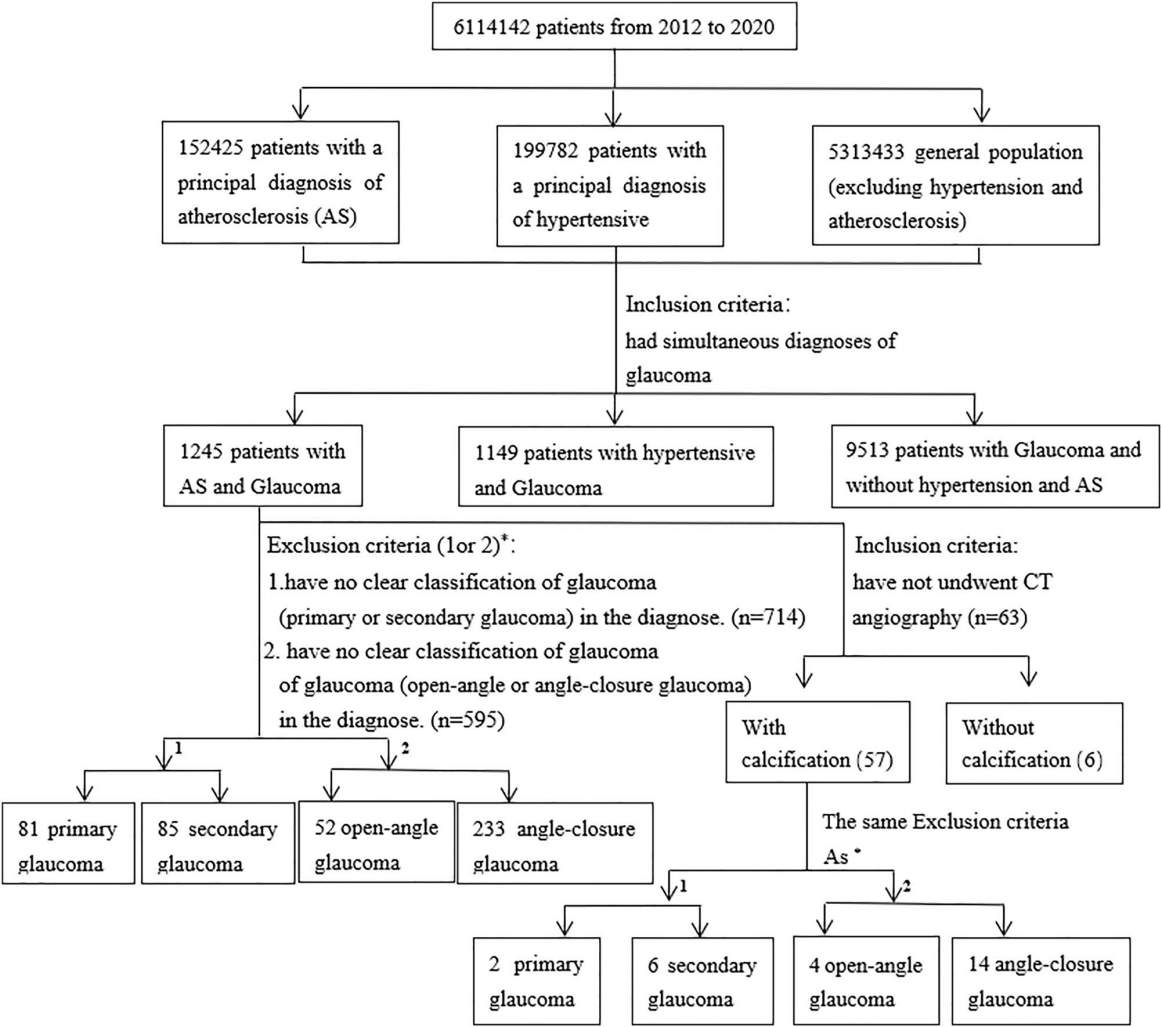
The analysis process of the morbidity of glaucoma in patients with AS.

In this study, patients with a principal diagnosis of AS or hypertension (excluding AS) and patients without hypertension or AS were selected, and the number of patients who also suffered from glaucoma in both groups was obtained by filtering diagnoses using glaucoma as the query term. The CT examination findings and diagnostic information of the patients with both AS and glaucoma were derived, and the incidence rates of the different types of glaucoma in the different groups were analyzed. The analysis process is described in Fig. 3.

Among 152,425 AS patients, 1245 patients were also diagnosed with glaucoma (8.17‰). The incidence of glaucoma in the general hypertensive population (excluding AS) was 5.75‰ (1149/199,782 patients), and the incidence of glaucoma in patients without hypertension or AS was 1.79‰ (9513/5,313,433 patients). The incidence rate of glaucoma in the AS patients was significantly higher than those in the hypertensive population and the general population (both *p* values between groups were below 0.001).

Among the 1245 AS patients with a simultaneous diagnosis of glaucoma, the data of 365 patients were analyzed as described above. Of the remaining 880 patients, 166 patients were specifically diagnosed with primary or secondary glaucoma. The remaining 714 patients were diagnosed with only glaucoma. Among the 166 patients, 81 (48.8%) had primary glaucoma, and 85 (51.2%) had secondary glaucoma. Among the 880 patients, 52 patients (18.2%) had open-angle glaucoma, and 233 (81.8%) patients had angle-closure glaucoma. The remaining 595 patients were diagnosed with only glaucoma. This observation indicated that AS patients with glaucoma were more likely to suffer from angle-closure glaucoma than from open-angle glaucoma.

Among the 880 patients with concurrent diseases, 63 patients underwent arterial CT angiography, including 45 cases of coronary artery angiography, 17 cases of head and neck angiography, and 11 cases of aorta angiography. Angiography examination showed that fifty-seven (90.5%) patients had varying degrees of arterial calcification. Among these 57 patients, 8 patients were diagnosed with primary glaucoma (2 patients or 25%) or secondary glaucoma (6 patients or 75%), and 18 patients were diagnosed with open-angle (4 patients or 22%) or angle-closure glaucoma (14 patients or 78%). This observation indicated that AS patients with arterial calcification mostly suffered from secondary glaucoma and angle-closure glaucoma.

The age composition of each group was also analyzed. A total of 951 (77.0%) of the 1245 patients with both AS and glaucoma (group 1) were more than 65 years old. A total of 58.7% (674/1149 patients) of the patients diagnosed with both hypertension (excluding AS) and glaucoma (group 2) and 31.5% (2999/9513 patients) of the patients with glaucoma in the general population (excluding hypertension and AS patients) (group 3) were more than 65 years old. Differences in the age compositions between group 1 and groups 2 and 3 were significant (*p*<0.01). The above observations suggested that AS complicated with glaucoma was more likely to occur in people more than 65 years old.

### Analyzing the potential therapeutic effect of MTZ on AS

To observe the therapeutic effect of MTZ on AS, we extracted the data of patients who had both glaucoma and AS and were treated with MTZ for glaucoma. These patients underwent biochemical tests before and after MTZ treatment. The patients took MTZ for two weeks and did not take any lipid-lowering drugs, such as statins, before biochemical testing. Finally, 14 patients who received MTZ and underwent biochemical testing 3 months later were selected from the Yiduyun database of Shandong Provincial Qianfoshan Hospital. Among the 14 patients, 9 showed decreased LDL levels after MTZ treatment**.** The average LDL level was 2.81 ± 0.61 mmol/L before treatment, and the average LDL level was 2.38 ± 0.58 mmol/L after treatment (*p* = 0.039). This observation indicated that MTZ decreased LDL levels while treating glaucoma. On the other hand, 5 patients (36%) had increased HDL levels after MTZ treatment. The average HDL level before treatment was 1.22 ± 0.28 mmol/L, and the average value after MTZ treatment was1.46 ± 0.37 mmol/L. This observation indicated that MTZ seemed to increase HDL levels following MTZ treatment for glaucoma, although the difference was not significant (*p* = 0.08). The lipoprotein changes before and after medication are shown in Table 2.

**Table 2:** The lipoprotein levels before and after medication.

No	patients	Age	Gender	lipoprotein	Before medication	After medication
1	94	62	Female	LDL	3.32	3.16
				HDL	0.78	0.85
2	375	29	Female	LDL	2.85	1.81
				HDL	1.11	1.75
3	565	57	Male	LDL	2.1	2.09
				HDL	0.77	0.76
4	605	73	Female	LDL	3.73	2.15
				HDL	1.33	1.5
5	639	59	Male	LDL	2.9	2.61
				HDL	1.32	1.25
6	685	66	Female	LDL	3.46	3.43
				HDL	1.45	1.34
7	861	68	Male	LDL	2.66	2.18
				HDL	1.71	1.34
8	948	70	Male	LDL	2.05	1.78
				HDL	1.04	0.83
9	297	80	Female	LDL	2.25	2.17
				HDL	2.17	1.79
10	385	54	Female	LDL	2.83	3.54
				HDL	1.35	1.23
11	455	67	Male	LDL	2.8	3.31
				HDL	1.24	0.83
12	657	82	Male	LDL	2.96	3.21
				HDL	1.37	1.45
13	933	65	Female	LDL	3.55	3.75
				HDL	1.5	1.76
14	719	49	Female	LDL	2.19	3.07
				HDL	1.35	1.31

## Discussion

In this study, medical information from databases of two hospitals was analyzed to evaluate the correlation between AS and glaucoma. The incidence of glaucoma (0.817%) in patients with AS was significantly higher than that in patients with general hypertension (excluding AS) (0.575%) and patients without hypertension and AS (0.179%) (both *p* values were below 0.001). Meanwhile, the incidence of AS (6.45%) in the patients with glaucoma was significantly higher than that in the patients with keratitis (2.46%) or scleritis (3.52%) and the population without ophthalmic records (1.92%) (all *p* values were below 0.001). Additionally, 79.5% of the patients with glaucoma complicated with AS were diagnosed with angle-closure glaucoma, and 80.9% of the patients with AS complicated with glaucoma were diagnosed with angle-closure glaucoma. This observation indicated that glaucoma patients with AS were more likely to suffer from angle-closure glaucoma. We also found that the incidence of glaucoma complicated with AS and AS complicated with glaucoma was higher in people older than 65 years, indicating that patients aged 65 and older are prone to both glaucoma and AS. Furthermore, CT angiography showed that patients with arterial calcification were more likely to develop both AS and glaucoma (> 90%).

Carbonic anhydrases (CAs), which catalyze the synthesis of HCO_3_^-^ from H_2_O and CO_2_, can be found in many organs and tissues^15,16^. The malfunction of CAs or the abnormal expression of their genes leads to the pathogenesis of various diseases^15,17–20^. CAs located in eyes are associated with glaucoma^6^. In our previous study, CA1, a subtype of the CA family, was found to be overexpressed in the synovial membrane in patients with ankylosing spondylitis^21^, and high CA1 expression promotes joint calcification, ossification and joint fusion by accelerating calcium carbonate deposition^22,23^. CA1 was also highly expressed in breast carcinoma tissues and in blood from patients with breast cancer, leading to calcification of the tumor tissues, inhibition of apoptosis, and promotion of tumor cell migration^24^. Recently, we found that CA1 was overexpressed in aortic atherosclerosis tissues. Based on the above facts, CA1 could be involved in systemic diseases with abnormal calcification and ossification. Because increased CA1 expression was detected in aortic atherosclerosis tissues and CA expression was also located in eyes with glaucoma, high CA1 expression or high enzymatic activity of carbonic anhydrase may be responsible for the strong association between AS and glaucoma.

In our previous study, we found that MTZ showed therapeutic and preventive effects on AS in a mouse model^11^. In the current study, 14 patients were screened from the Yiduyun Database who had both AS and glaucoma, treated with MTZ for glaucoma and underwent serum lipid tests. Among them, 9 of the patients showed decreased LDL levels within 3 months after treatment (2.81 ± 0.61 mmol/L vs. 2.38 ± 0.58 mmol/L, *p* = 0.039). It has been well established that a high level of LDL is a key risk factor for AS^25,26^, and HDL correlates inversely with cardiovascular risk^10,27,28^. However, due to the small number of treated cases with available follow-up lipid profiles and since decreasing LDL cholesterol cannot be considered a reversal of atherosclerosis, we cannot conclude that MTZ treatment may reduce LDL levels in glaucoma patients and have a therapeutic effect on AS.

In conclusion, this study found an intrinsic link between AS and glaucoma. The results suggested that patients with glaucoma have a higher risk of developing AS than patients with other eye diseases, and patients with AS have a higher risk of developing glaucoma than those with hypertension or the general population.

## Data Availability

The authors will publish the dataset in a publicly available online repository 1 year after the publication of this manuscript.
